# Co-endemicity of *Plasmodium falciparum* and Intestinal Helminths Infection in School Age Children in Rural Communities of Kwara State Nigeria

**DOI:** 10.1371/journal.pntd.0003940

**Published:** 2015-07-29

**Authors:** Ayodele Adedoja, Bukola Deborah Tijani, Ajibola A. Akanbi, Taiwo A. Ojurongbe, Oluwaseyi A. Adeyeba, Olusola Ojurongbe

**Affiliations:** 1 Department of Medical Microbiology and Parasitology, Ladoke Akintola University of Technology, Osogbo, Nigeria; 2 Department of Medical Microbiology and Parasitology, University of Ilorin Teaching Hospital, Ilorin, Nigeria; 3 Department of Medical Microbiology and Parasitology, University of Ilorin, Ilorin, Nigeria; 4 Department of Mathematical and Physical Sciences, Osun State University, Osogbo, Nigeria; University of Cambridge, UNITED KINGDOM

## Abstract

**Background:**

Malaria and intestinal helminths co-infection are major public health problems particularly among school age children in Nigeria. However the magnitude and possible interactions of these infections remain poorly understood. This study determined the prevalence, impact and possible interaction of *Plasmodium falciparum* and intestinal helminths co-infection among school children in rural communities of Kwara State, Nigeria.

**Methods:**

Blood, urine and stool samples were collected from 1017 primary school pupils of ages 4–15 years. Stool samples were processed using both Kato-Katz and formol-ether concentration techniques and microscopically examined for intestinal helminths infection. Urine samples were analyzed using sedimentation method for *Schistosoma haematobium*. *Plasmodium falciparum* was confirmed by microscopy using thick and thin blood films methods and packed cell volume (PCV) was determined using hematocrit reader. Univariate analysis and chi-square statistical tests were used to analyze the data.

**Results:**

Overall, 61.2% of all school children had at least an infection of either *P*. *falciparum*, *S*. *haematobium*, or intestinal helminth. *S*. *haematobium* accounted for the largest proportion (44.4%) of a single infection followed by *P*. *falciparum* (20.6%). The prevalence of malaria and helminth co-infection in the study was 14.4%. Four species of intestinal helminths were recovered from the stool samples and these were hookworm (22.5%), *Hymenolepis* species (9.8%), *Schistosoma mansoni* (2.9%) and *Enterobius vermicularis* (0.6%). The mean densities of *P*. *falciparum* in children co-infected with *S*. *haematobium* and hookworm were higher compared to those infected with *P*. *falciparum* only though not statistically significant (p = 0.062). The age distribution of both *S*. *haematobium* (p = 0.049) and hookworm (p = 0.034) infected children were statistically significant with the older age group (10–15 years) recording the highest prevalence of 47.2% and 25% respectively. Children who were infected with *S*. *haematobium* (RR = 1.3) and hookworm (RR = 1.4) have equal chances of being infected with *P*. *falciparum* as children with no worm infection. On the other hand children infected with *Hymenolepis spp*. (p<0.0001) are more likely to be infected with *P*. *falciparum* than *Hymenolepis spp*. uninfected children (RR = 2.0)

**Conclusions:**

These findings suggest that multiple parasitic infections are common in school age children in rural communities of Kwara State Nigeria. The *Hymenolepis* spp. induced increase susceptibility to *P*. *falciparum* could have important consequences on how concurrent infections affect the expression or pathogenesis of these infections.

## Author Summary

Malaria, schistosomiasis and intestinal helminths are parasitic diseases responsible for high morbidity and mortality in most tropical areas of the world. These parasitic diseases are common in Nigeria where people are co-infected with more than one parasite. This study investigate the prevalence and impact of these parasite co-infections in school age children in rural communities of Kwara State Nigeria. The result shows that over 61.0% of the school children had at least an infection of either *P*. *falciparum*, *S*. *haematobium*, or intestinal helminths with *S*. *haematobium* and *P*. *falciparum* accounting for the largest proportion of a single infection with 44.4% and 20.6% respectively. Children who were infected with *S*. *haematobium* (RR = 1.3) and hookworm (RR = 1.4) have equal chances of being infected with *P*. *falciparum* as children when compared with children with no infection. On the other hand children infected with *Hymenolepis* spp. (p<0.0001) have a higher chance of being infected with *P*. *falciparum* when compared to uninfected children (RR = 2.0). These findings suggest that *Hymenolepis* spp. may be associated with increase susceptibility to *P*. *falciparum* in this population of Nigeria children.

## Introduction

Malaria and helminth infection are the most important public-health problems affecting children in Sub-Saharan Africa [1]. It is estimated that over a third of the world's population, mainly those individuals living in the tropics and subtropics, are infected by parasitic intestinal helminths or one or more of the species of *Plasmodium* [2]. Malaria caused by *Plasmodium falciparum* is responsible for a high burden of disease and a loss of growth in endemic countries estimated to be as high as 1.3% of gross domestic product per year [3]. Helminths infections on the other hand though not as deadly as *falciparum* malaria infection are associated with high morbidity and mortality occurring in sub-Saharan Africa [4]. Different environmental factors and host related activities influences the distribution of the different Intestinal helminths that includes nematodes trematodes and cestodes which make up these multiple infections [5,6]. These factors include poverty, environmental contamination with infected faeces containing helminth eggs, water bodies, inadequately cooked foods, lack of effective preventive measures [7] and immunity of the host [8].

Malaria and helminths infections are widespread and they both have similar geographical and overlapping distribution in developing countries with the major consequence of the co-infection being anaemia [9,3]. The major soil transmitted helminths (*Ascaris lumbricoides*, hookworm and *Trichuris trichiura*), coupled with schistosomiasis are responsible for more than 40% of the worldwide morbidity from all tropical infections, excluding malaria [10]. Children co-infected with these parasites have been shown to have hampered cognitive and physical development that leads to reduced learning and school achievements and are also prone to increased susceptibility to other infections [11,12]. Concomitant parasitic infections could induce modifications of the specific immune response to each pathogen and thus leading to modification of clinical expression [13]. Studies have shown that helminths can either protect [14,15] or worsen [16,17] malaria severity and young children from rural areas are the most affected.

In Nigeria, *falciparum* malaria and helminths infections are reportedly endemic and pose a significant health problem among children [18,19]. They are particularly more prevalent in rural communities and are closely associated with poverty [20,21]. Studies have shown that individuals co-infected with more than one parasite species are at risk of increased morbidity as well as at a risk of developing frequent and more severe disease due to interactions among the infecting parasite species [22,23]. Despite existence of contrasting evidence on the interaction of helminth and malaria infection, more results have pointed to the fact that individuals infected by helminth are more likely to develop malaria than helminth free individuals [24,17]. Considering the limited number of studies on interactions between malaria and helminth co-infections in human populations, the present study being the first study in this study area was undertaken to investigate the prevalence and impact of *P*. *falciparum* malaria parasitaemia and helminth co-infection in school children living in a setting where malaria is endemic. The results of this study could provide valuable information to local health authorities for improving existing control strategies.

## Methods

### Ethical considerations

The study was approved by the Kwara State ministry of Health Ethical Committee (MOH/KS/777/41). Before sample collection meetings were held with community leaders, teachers and community members. The objectives of the study including the study procedures, samples to be taken, study benefits, potential risks and discomforts were explained. Informed consent for all children who participated in the study was sought from parents and legal guardians after they have been clearly informed about the study. Parental consent given from parents/guardians were verbal. The ethical committee approved the use of oral consent as most parents were illiterates. Children were also requested to give consent and were informed of their right to refuse to participate in the study and to withdraw at any time during the study without jeopardizing their right of access to other health services. Consent was recorded as ‘yes’ or ‘no’ on an individual form designed for sample collection. The pains and the precautions of procedures such as collection of blood samples were fully explained to parents and children. Newly opened needle and syringe were used for each child. Before samples were collected, demographic data such as sex, age, weight, height and name of subject were recorded. Subjects diagnosed with malaria or helminths were treated appropriately free of charge according to national guidelines. Identification numbers were used instead of children names and information collected were kept confidential.

### The study area, population and design

This cross-sectional study was conducted among primary and secondary schools in Pategi and Lafiagi communities in Nigeria. Pategi and Lafiagi are located in Pategi and Edu Local Government Areas (LGAs) in Kwara State, Nigeria. Population of Pategi is 110,852 people while that of Lafiagi is 102,780 people [25]. Both communities fall into stable malaria transmission zone where malaria is present throughout the year with a marked increase during the rainy season which normally runs from April to September. The towns stands on higher level and the soil can be described as well drained, moderately leached and with moderate humus content. Major occupations include farming (rice farming), fishing and petty trading. The study was carried out from October 2012 to May 2013, which spanned through the dry and rainy season. All school children who were willing to be part of the study were included in the study.

### Sampling procedure

#### Detection and quantification of malaria parasites

About 2 ml of blood samples were collected by venous blood for the determination of *P*. *falciparum* parasitemia. Thick blood films were prepared, air dried, Giemsa-stained, and observed under the microscope for identification and quantification of malaria parasites. Malaria parasites were counted against 200 leukocytes, and counts were expressed as the number of parasites per microliter of blood, assuming an average leukocyte count of 8,000 cells/μl of blood [26].

#### Determination of packed cell volume

For Packed Cell Volume (PCV), micro hematocrit tubes filled with blood were centrifuged in a micro-hematocrit rotor for 5 minutes at 10,000g. The tubes were placed in the micro-hematocrit reader and children with PCV values ≤ 31% were considered as anaemic, which was further classified as mild (21–30%), moderate (15–20%), or severe (≤15%) [27].

#### Detection and quantification of helminths

Clean plastic containers were distributed for both stool and urine collection, and instructions were given for proper collection. Instructions were given to collect only terminal drops of urine The formol-ether concentration technique and Kato-Katz thick smear technique were used for quantitative determination of helminths ova [28]. Stool samples were processed within 12 hours of collection and examined microscopically within 1 hour of preparation to avoid missing hookworm ova. The intensity of infection was expressed as the number of eggs per gram of faeces (epg). Water—or urine-contaminated stools were rejected. The number of helminth eggs were counted and multiplied by 24 in order to quantify the number of eggs per gram (epg) of faeces. To ensure consistency of the result and as a form of quality control, 20% of the slides were randomly selected and read again [29].

Urine samples were analyzed using sedimentation method. Samples were left to stand on the bench for about 30 minutes. Afterwards the topmost part of the urine was discarded leaving about 10 ml in the bottle. The contents of each bottle was mixed thoroughly with the sediment and was transferred into a 20 ml centrifuge tube. The tubes were then centrifuged at 1000 r/min for 2 min. The supernatant was discarded and the residue was put on a clean glass slide and examined under 10X objective lens of the microscope. Intensity of infection was estimated as number eggs per 10ml of urine.

### Statistical analysis

Data were double-entered and cross-checked in Microsoft Excel version 2013. Statistical analysis was done using SPSS version 16 for windows. For univariate analysis, frequencies were compared using the Fisher's exact tests. Prevalence of malaria, helminth and co-infection and gender were compared using χ^2^ tests. Relative Risk (RR) were calculated for risk estimation between *P*. *falciparum* and helminths. Logistic regression was carried out to investigate the relationship between anemia and occurrence of parasitic infection in the study population. P-value ≤ 0.05 was considered significant during the analysis

## Results

A total of 1017 blood, urine and stool samples collected from primary school children aged 4 to 15 years were microscopically investigated in other to determine the prevalence and impact of *S*. *haematobium*, intestinal helminths and malaria co-infection. The mean age of the study population was 9.52 ± 1.904 years while the mean height was 124.19 ± 2.54 cm and the mean weight was 25.34 ± 4.26 kg.

The prevalence and intensities of all the parasites seen in the blood, stool and urine samples of the study subjects are shown in Table 1. Overall, 61.2% of all the school children had at least an infection of either *P*. *falciparum*, *Schistosoma haematobium*, hookworm, *Hymenolepis* specie or *Enterobious vamicularis*. *S*. *haematobium* accounted for the largest proportion (44.4%) of a single infection followed by *P*. *falciparum* parasitaemia (20.6%). Hookworm and *Hymenolepis* spp. had a prevalence of 22.5% and 9.8% respectively while *E*. *vermicularis* had the lowest prevalence of 0.6%. The intensities of infection expressed as geometric mean parasite count of positive samples is shown in Table 1.

**Table 1:** Overall prevalence and infection intensities (expressed as geometric mean parasite count of positive samples) of parasitic infections in school children in Kwara State Nigeria.

Characteristics n = 1017	*P*. *falciparum*	*S*. *haematobium*	Hookworm	*Hymenolepis spp*	*E*. *vermicularis*
No. Infected	209	452	229	100	6
Prevalence (%)	20.6	44.4	22.5	9.8	0.6
Geo Mean Parasite density	316.5p/μl	52.3/ml	990.3/g	476.5/g	200/g
Maximum density	14040/μl	300/ml	3000/g	2200/g	200/g

The mean age of the study population is 9.5 ± 1.9 years. Table 2 shows the distribution of parasitic infection by age in the studied population. For *P*. *falciparum* parasitaemia, the youngest age group (4–9 years) had the highest prevalence of 22.7% but the difference was not statistically significant (p = 0.14). The distribution of *S*. *haematobium* by age was slightly significant (p = 0.049) with the older age group (10–15 years) recording the highest prevalence (47.2%). Distribution of hookworm by age also showed a significant difference (p = 0.034) with the older age group recording the highest prevalence (25%) of infection. No significant difference was observed in the age distribution of *Hymenolepis* spp. and *E*. *vermicularis* in the study population

**Table 2:** Prevalence of single parasitic infection among children by age.

Age group	No. Exam	Prevalence of single infection (%)				
		PF	SH	HW	HN	EV
**4–9**	445	101 (22.7)	182 (40.9)	86 (19.3)	45 (10.1)	3 (0.7)
**10–15**	572	108 (18.9)	270 (47.2)	143 (25.0)	55 (9.6)	3 (0.5)
**Total**	1017	209 (20.6)	452 (44.4)	229 (22.5)	100 (9.8)	6 (0.6)
**p-value**		0.14	0.049*	0.034*	0.83	0.73

*Significant p<0.05

Key: PF = *P*. *falciparum*; SH = *S*. *haematobium*; HW = Hookworm; HN = *Hymenolepis* species, EV = *Enterobious vermicularis*

The distribution of parasitic infection according to gender among the school children is presented in Table 3. A total of 519 (51.0%) males and 498 (49.0%) females were enrolled in the study. In all, males were generally more infected with all the intestinal helminths and the difference was not significant. Similarly for *P*. *falciparum* parasitaemia, female (21.1%) were slightly more infected than males (20.0%) but the difference was not statistically significant (p = 0.69).

**Table 3:** Prevalence of single parasitic infection among children by sex.

Sex	No. Exam	Prevalence of single infection (%)				
		PF	SH	HW	HN	EV
**Male**	519	104 (20.0)	244 (47.0)	130 (25.0)	60 (11.6)	4 (0.8)
**Female**	498	105 (21.1)	208 (41.8)	99 (19.9)	40 (8.1)	2 (0.4)
**Total**	1017	209 (20.6)	450 (44.3)	229 (22.5)	102 (10.0)	6 (0.6)
**p-value**		0.69	0.10	0.05	0.07	0.687

Key: PF = *P*. *falciparum*; SH = *S*. *haematobium*; HW = Hookworm; HN = *Hymenolepis* species, EV = *Enterobious vermicularis*

The prevalence of polyparasitism based on age and sex in the study population is shown in Table 4. Polyparasitism was generally higher among the male children but the difference was not statistically significant. No significant difference was also observed when total polyparasitism was compared between the age groups (p = 0.20)

**Table 4:** Prevalence of children infected with double infection by age and sex.

Age group	Gender	No. Exam	PF+SH(%)	PF+HW(%)	PF+HN(%)	SH+HW(%)	SH+HN (%)	HW+HN (%)
**4–9**	Male	221	28 (12.7)	13 (5.9)	12 (5.4)	34 (15.4)	14 (6.3)	8 (3.6)
	Female	225	26 (11.6)	12 (5.3)	9 (4.0)	26 (11.6)	3 (1.3)	3 (1.3)
	Total	446	54 (12.1)	25 (5.6)	21 (4.7)	60 (13.5)	17 (3.8)	11 (2.5)
**10–15**	Male	298	29 (9.7)	22 (7.4)	9 (3.0)	58 (19.5)	16 (5.4)	11 (3.7)
	Female	274	23 (8.4)	14 (5.1)	12 (4.4)	46 (16.8)	16 (5.9)	9 (.3)
	Total	572	52 (9.1)	36 (6.3)	21 (3.7)	104 (18.2)	32 (5.6)	20 (3.5)
**Overall**	Male	519	57 (11.0)	35 (6.7)	21 (4.0)	92 (17.7)	30 (5.8)	19 (3.7)
	Female	498	49 (9.8)	26 (5.2)	21 (4.2)	72 (14.5)	20 (4.0)	13 (2.6)
	Total	1017	106 (10.4)	61 (6.0)	41 (4.0)	164 (16.1)	60 (5.9)	32 (3.1)

Key: PF = *P*. *falciparum*; SH = *S*. *haematobium*; HW = Hookworm; HN = *Hymenolepis* species, EV = *Enterobious vermicularis*

The association between the presence of malaria parasitaemia and intestinal helminths among the study subjects is shown in Table 5. There was a statistically significant association between *P*, *falciparum* and *S*. *haematobium* (p = 0.04), *P*. *falciparum* and hookworm (p = 0.01) with a Relative Risk of 1.3 and 1.4 respectively. Children who were infected with *S*. *haematobium* (RR = 1.3) and hookworm (RR = 1.4) have equal chances of likely being infected with *P*. *falciparum* as children with no worm infection. On the other hand, children exposed to *Hymenolepis* spp. (p<0.0001) are more likely to be infected with *P*. *falciparum* than *Hymenolepis* spp. uninfected children (RR = 2.0)

**Table 5:** Associations between helminths and *Plasmodium falciparum* infection, according to helminth species.

Helminth Infection	*P*. *falciparum* Infection (%)		Relative Risk (CI)	p-value
	Present n = 209	Absent n = 808		
***S*. *haematobium***	106 (50.7)	346 (42.8)	1.3 (1.0–1.6)	0.04
**Hookworm**	61 (29.2)	168 (20.8)	1.4 (1.0–1.8)	0.01
***Hymenolepis spp***	42 (20.1)	58 (7.2)	2.0 (1.6–2.7)	<0.0001

A logistic regression analysis was carried out to determine the occurrence of anemia among the school children using the single and double parasitic infection as predictors (Table 6). *Plasmodium falciparum* (P = <0.001), *S*. *haematobium* (p < 0.001) and hookworm (p < 0.001) infections showed strong significant association with anemia. Also the occurrence of *P*. *falciparum* + *S*. *haematobium* (P = <0.001), *P*. *falciparum* + hookworm (p = 0.004), *S*. *haematobium* + hookworm (P = <0.001) and *P*. *falciparum* + *Hymenolepis* spp. (P = <0.001) co- infections were strong predictors of the occurrence of anemia in the study population

**Table 6:** Logistic regression predicting the occurrence of anemia in relation to parasitic infection among the school children.

Predictor	No. positive (%)	Odds	Wald χ^2^	p-value	Odds Ratio
PF	209 (20.6)	1.417	61.697	<0.001*	4.126
SH	450 (44.3)	0.907	32.363	<0.001*	2.476
HW	229(22.5)	1.601	82.657	<0.001*	4.957
HN	102 (10.0)	0.492	4.025	0.045*	1.636
PF+SH	106 (10.4)	1.009	15.109	<0.001*	2.744
PF+HW	61 (6.0)	1.203	8.466	0.004*	3.328
SH+HW	164 (16.1)	1.724	73.282	<0.001*	5.606
PF+HN	41(4.0)	1.562	15.422	<0.001*	4.768
SH+HN	60 (5.9)	-0.421	0.804	0.370	0.656
HW+HN	32 (3.1)	0.680	1.390	0.238	1.973

*Significant at p<0.05

Key: PF = *P*. *falciparum*; SH = *S*. *haematobium*; HW = Hookworm; HN = *Hymenolepis* species, EV = *Enterobious vermicularis*

**Fig 1:**
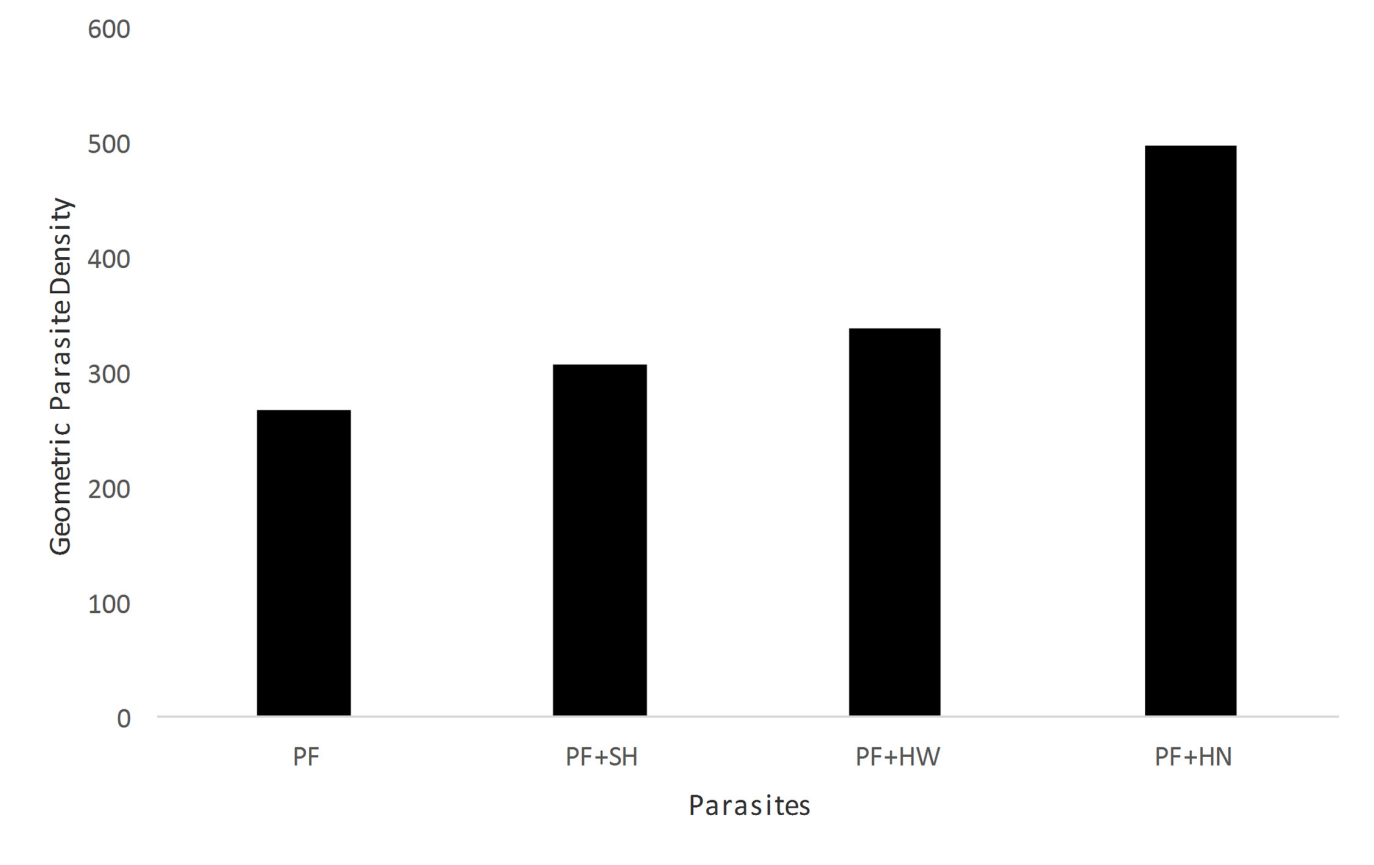
Geometric parasite density per microlitre of blood by type of infection. PF vs PF+HN = p< 0.001; PF + HW Vs PF + SH = p< 0.001;Key: PF = *P*. *falciparum*; SH = *S*. *haematobium*; HW = Hookworm; HN = *Hymenolepis* species.

Children infected exclusively with *P*. *falciparum* alone had the lowest geometric mean parasite density (GMPD) per microliter of blood in the study population. Significantly high GMPD was observed among children co-infected with *P*. *falciparum* + *Hymenolepis* spp. as compared to those with *P*. *falciparum* alone. Also *P*. *falciparum* + hookworms and *P*. *falciparum* + *S*. *haematobium* co-infections also recorded a significantly higher GMPD in comparison to *P*. *falciparum* alone (p < 0.001) (Fig 1).

**Fig 2:**
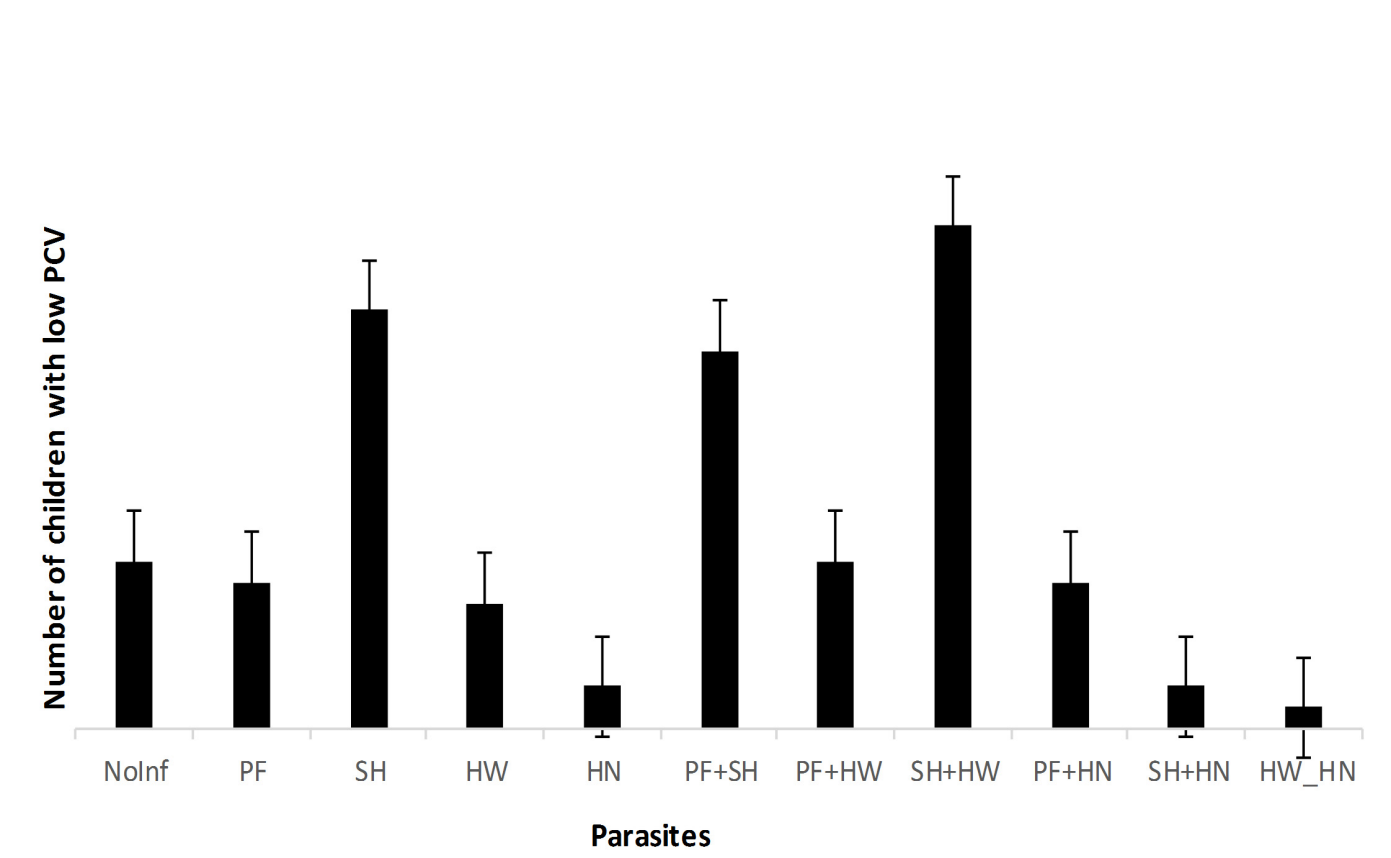
The proportion of children with anemia by type of infection. Key: PF = *P*. *falciparum*; SH = *S*. *haematobium*; HW = Hookworm; HN = *Hymenolepis* species.

Fig 2 showed the proportion of children with low PCV with respect to the different parasites. Children infected with *S*. *haematobium* alone, co-infection of *P*. *falciparum* and *S*. *haematobium*, co-infection of *S*. *haematobium* and hookworm had significantly higher proportion of children with low PCV when compared to children without infection. None of the children in the study population presented with severe anemia while the prevalence of moderate and mild anemia was 1.2% and 30.0% respectively.

## Discussion

This study suggests that underlying infection with *Hymenolepis* spp. may increase the chance of being infected with *P*. *falciparum* and also increase the geometric mean parasite count of *P*. *falciparum*. The implication of this is that *Hymenolepis* spp. modestly increases the degree of susceptibility against *P*. *falciparum* malaria among school children in rural communities of Ilorin, Nigeria. To the best of our knowledge, this work provides the first report of the prevalence of *Hymenolepis* spp. and its association to *P*. *falciparum* infection. *Hymenolepis* spp. is not a frequently encountered helminth infection in Nigeria. Previous authors have reported a higher prevalence of 18.3% in the 80s in the Niger Delta area of the country [30] while lower prevalence has been reported in other parts of the country [31]. *Hymenolepis* spp. often remain asymptomatic and severe infections can occur in individuals with more than 20,000 eggs per gram of feces. Symptoms of severe diseases include enteritis with or without diarrhea, abdominal pain, and loss of appetite. Children may experience more severe intestinal symptoms, which can include epilepsy, probably due to the toxic action that the products of the inflammation cause [32]. Further work on the immunological interaction will be needed to further elucidate the impact of this parasite in this population.

A near to equal association was observed between *P*. *falciparum* infection and *S*. *haematobium* infection and *P*. *falciparum* infection and hookworm infection. In many previous studies, observation for the interaction between *P*. *falciparum* infection and *S*. *haematobium* infection had yielded contrasting reports. While some studies reported increased susceptibility to *P*. *falciparum* infection in the presence of *S*. *haematobium* [16] others have shown protective effect of *S*. *haematobium* on *P*. *falciparum* [15]. These contrasting results have left important questions unanswered about the biological associations between *P*. *falciparum* and helminths. Explanations for these contrasting results have been given in terms of immunonological interactions and microgeographical variation [33]. The rates of co-infection may be influenced not only on chance, but also on the spatial distribution of environmental conditions that favor the transmission of multiple species [7,34]. Also immunological interactions and common factors that affect genetic susceptibility or host behavior can also influence co-infection rate [34]. In all, this study demonstrated that malaria, schistosomiasis and soil-transmitted helminth infections are of public health importance among primary schools pupils in Pategi and Lafiagi, Kwara State, Nigeria and co-infections of these parasites are common and interactions could have a negative effect on the infected school children. The findings of *P*. *falciparum*, schistosomiasis and helminth co-infection observed in this study are supported by other previous studies in Sub-Saharan Africa [27,22].

The most prevalent parasites in this study were *S*. *haematobioum*. *P*. *falciparum*, hookworm and *Hymenolepis* spp. The prevalence of urinary schistosomiasis (44.4%) reported in this study suggests that Pategi and Lafiagi LGAs of Kwara State are endemic for urinary schistosomiasis. Earlier study in Bida Niger State, Nigeria a community that shares both socio-cultural and geographical attributes with Pategi and Lafiagi showed high prevalence of urinary schistosomiasis [35]. The prevalence of co-infection of malaria and helminths (14.4%) observed is an indication of high polyparasitism among school children in the study area. Co-infection of *P*. *falciparum* with *S*. *haematobium* was the highest, followed by *P*. *falciparum* and hookworm. Both *falciparum* malaria and helminths have similar geographical distribution and it is estimated that over a third of the world's population, mainly those living in the tropics and subtropics are infected [3]. Factors that may support co-infections which were present in the area of study include the abundant presence of mosquito and snail vectors, temperature, humidity, stagnant bodies of water, bushy environment, unhygienic environment and poor drainage. Others include poor sanitary disposal, open air defecation, usage of night soil as fertilizer in farming, barefoot walking which can predispose subjects to penetration of infective larvae of hookworm.

The observed prevalence of both *S*. *haematobium* and hookworm are significant with respect to age, with the older age group (10–15 years) being more infected. This reflects the fact that older age groups are involved in more water activity contact and also have higher duration of exposure to infection of other soil transmitted helminths. For *P*. *falciparum* parasitaemia, the prevalence was higher among the younger age group. It was previously observed that the majority of malaria infections in individuals living in endemic regions are asymptomatic with the young children bearing the highest burden of disease and carrying asymptomatic infections for most of the time [36,18]. The decline of *P*. *falciparum* parasite prevalence with age, observed in this study is in agreement with our previous study [18] and it is the characteristic of both symptomatic and asymptomatic falciparum infection in endemic regions [37,38]. *Ascaris lumbricoides* which was one of the commonest intestinal parasite in Nigeria was not detected in the current study. This observation has been previously made by some authors who have reported this specie to be rare in some areas in Sub-Saharan Africa [22,4].

About 39% of the children in the study were classified as anemic with majority being moderately anemic. Many studies have reported anemia as one of the major health problems resulting from malaria [39,40], hookworm and *S*. *haematobium* [41,4] infection in Nigeria. The highest proportion of school children with low PCV was observed among those co-infected with hookworm and schistosomiasis followed by schistosomiasis alone and *P*. *falciparum* schistosomiasis co-infected school children. Also the logistic regression analysis predicts that the occurrence of anemia among the school children is strongly influenced by the presence of single or co-infection of *P*. *falciparum*, hookworm and *S*. *haematobium*. These observations further confirms previous reports of a positive relationship that exist between parasitic infections and anemia and also the possible synergistic interaction of these parasites as a strong etiology of anemia [40,41,42].

Higher mean geometric parasite density of *P*. *falciparum* was observed in co-infection with helminths when compared to *P*. *falciparum* alone. This observation has been previously reported by other authors [43,44]. Higher mean geometric parasite density was observed in individuals simultaneously co-infected with both *P*. *falciparum* and *S*. *haematobium*, *P*. *falciparum* and hookworms or *P*. *falciparum* and *Hymenolepis* spp. It thus appears that *S*. *haematobium*, hookworm or *Hymenolepis* spp. are associated with higher prevalence of *P*. *falciparum* in this population of Nigeria children.

There is an urgent call by WHO and its collaborating partners to scale up interventions to control and eliminate Neglected Tropical Diseases (NTDs) in the WHO African region. Schistosomiasis and soil transmitted helminths are among the NTDs targeted for elimination. In other for this to materialize, the affected areas must be identified and its burden must be assessed. This remains a crucial and urgent roadmap in other to achieve complete control and elimination of the NTDs in Africa. This study is therefore important for policy makers and stakeholders in the control of NTDs in Africa.

Conclusively, high prevalence of co-infection of *P*. *falciparum*, *S*. *haematobium* and intestinal helminths was observed among school children in Pategi and Lafiagi communities of Kwara State Nigeria. This is the first study to document the prevalence of these co-infections and also highlighting the impact of *Hymenolepis* spp. and *P*. *falciparum* co-infection among children in the middle belt of Nigeria. The effort of Kwara State Government in reducing the morbidity caused by *S*. *haematobium* and intestinal helminths through yearly mass deworming with Albendazole and praziquantel treatment should be complimented with adequate health education. Toilets should be provided in the schools with water for hand washing and personal hygiene enforced in other to create attitudes that will break the cycle of this infections.

## Supporting Information

S1 Checklist STROBE checklist. (DOC)
